# Maternal and newborn healthcare practices: assessment of the uptake of lifesaving services in Hoima District, Uganda

**DOI:** 10.1186/s12884-020-03385-x

**Published:** 2020-11-11

**Authors:** Geoffrey Babughirana, Sanne Gerards, Alex Mokori, Elisha Nangosha, Stef Kremers, Jessica Gubbels

**Affiliations:** 1 NUTRIM School of Nutrition and Translational Research in Metabolism, Department of Health Promotion, Maastricht University, Maastricht, the Netherlands; 2 Independant Scholar, Kampala, Uganda; 3 World Vision International, Hoima, Uganda

**Keywords:** Maternal and newborn health, Goal-oriented ANC, Essential newborn care, Male involvement and quality of care

## Abstract

**Background:**

The current maternal mortality ratio in Uganda is 336 maternal deaths per 100,000 live births. Infant mortality is 43 deaths per 1000 live births, with 42% of the mortality occurring during the neonatal period. This might be related to a weak health system in the country. This study aimed at assessing the uptake of lifesaving services during pregnancy and childbirth in Hoima District, Uganda.

**Methods:**

The study used a cross-sectional quantitative design among 691 women with a child under 5 years. Households were randomly sampled from a list of all the villages in the district with the ENA for SMART software using the EPI methodology. Pre-coded questionnaires uploaded in the Open Data Kit were used for data collection. The data was cleaned and analysed using MS Excel and SPSS software. Descriptive results are presented.

**Results:**

Of the 55.1% women attending at least four antenatal care (ANC) visits, only 24.3% had the first ANC within the first trimester. Moreover, ANC services generally was of poor quality, with only 0.4% meeting all the requirements for quality of ANC service. The highest contributors to this poor quality included poor uptake of iron-folic acid (adherence 28.8%), the six-required birth preparedness and complication readiness items (13.2%), and recognition of the seven danger signs of pregnancy (3.0%). Adherence to the seven essential newborn care actions was very low (0.5%), mainly caused by three practices: initiating breastfeeding within 1 h (59.9%), lack of postnatal care within 24 h (20.1%), and failure to recognize the 6 danger signs of the newborn (2.4%). Only 11.1% of the males participated in all maternal and newborn care requirements, by encouraging women to seek healthcare (39.9%), accompanying them to healthcare (36.9%), and HIV counselling and support services (26.2%).

**Conclusion:**

The study reveals poor maternal and newborn practices throughout the continuum of care, from ANC and skilled birth attendance to newborn care during childbirth. With such poor results, it is not surprising that Hoima is sixth of 10 districts that have the highest numbers of deaths due to maternal mortality in Uganda.

## Background

In Uganda, around 2% of women die from maternal causes. With the current maternal mortality ratio of 336 maternal deaths per 100,000 live births, many women die from pregnancy and childbirth-related complications [1]. Uganda’s infant mortality currently stands at 43 deaths per 1000 live births, therefore 1 in 23 children dies before reaching their first birthday, with 42% of the mortality occurring during the neonatal period [2, 3].

In Uganda, 97% of pregnant women attend at least one antenatal care (ANC) visit. However, 60% of women in Uganda complete at least 4 out of the 8 recommended visits, and only 29% have their first visit during the first trimester [2, 3]. Even though 74% of childbirths in Uganda are attended by skilled health personnel, and 44% of women do not receive postnatal care (PNC) within 2 days after childbirth [2]. This implies that these women do not have the opportunity of childbirth and postnatal care during the first week of life, resulting in the large number of newborns lost during this period [4]. This is further exacerbated by the fact that in many cases, midwives’ positions in public health facilities are not filled, meaning that skilled providers of ANC and safe childbirth are not available [5].

During the first week of a newborn’s life, appropriate feeding, effective clean dry cord care and optimal thermal protection are lifesaving practices [6, 7]. In Hoima, only 31% of the women practiced appropriate clean dry cord care for the newborn, and 39.5% could not practice appropriate feeding [8], which could be due to insufficient preparation for the arrival of the newborn during ANC. For example, knowledge of clean dry cord care before childbirth was a very important predictor of the actual practice [8].

Predictors of optimal breastfeeding include staying more than 1 day in a health facility (HF), knowledge about correct positioning and attachment, and receiving support for earlier initiation of breastfeeding. The encouragement and practice of breastfeeding as the most optimal practice is linked to the survival of the newborn [6]. In Uganda, almost every child born has the opportunity to be breastfed (98%), with the 2% consisting of HIV affected mothers deciding not to breastfeed, and mothers who die during childbirth [6]. The challenge arises with practices related to the initiation and sustaining of breastfeeding [2]. For example, even though Uganda is a breastfeeding country, up to 35% of the women are not able to initiate breastfeeding within 1 h of giving birth, which normally contributes to sustained breastfeeding [4]. Late initiation of breastfeeding is attributed to poor practices by the skilled health personnel and community cultural practices against colostrum [4].

Male involvement is another factor that plays a role in pregnancy and essential newborn care and can influence behaviours at household and community level. Men can provide substantial support to women in order to overcome community-generated barriers to accessing pregnancy and childbirth health services [9]. Paternal support therefore contributes to the normalisation of pregnant women’s care-seeking behaviour [10]. Male partners can take on health-promoting behaviours at home, a process potentially encouraging others to adopt such behaviours as well, resulting in improved household nutrition and hygiene practices. Interventions to increase male involvement in pregnancy and essential newborn care have also been linked to improved couple relationships, including improved communication and shared decision-making, which in turn lead to positive health and care-seeking outcomes [11].

The current study aimed at assessing pregnancy and essential newborn care practices in Hoima District, Uganda. It focused on the household level and addressed the following sub-objectives in comparison to the required Ugandan targets: 1) uptake and quality of ANC services, 2) essential newborn care (ENC), and 3) level of male involvement in maternal and newborn care.

## Methodology

### Study area

The study was carried out in Hoima District of Uganda, in Bunyoro sub-region [12]. In 2012, the mid-year district population was estimated at 548,800 [13]. It is made up of 13 sub-counties, most of which are rural, with the urban centre based in the Hoima municipality, which is also the main trading centre of the district. A total of 31.9% of adults over 18 years old are illiterate, with 63% of the working population involved in agriculture and fishing.

### Study design and population

The study was a cross-sectional quantitative study. Women who had a child aged 0–59 months were included in the study. Once the woman gave an affirmative response about having a child aged 0–59 months, only those who consented participated in the study. The study enrolled 691 women, and women with multiple children within the selected age range were included only once into the study.

### Procedures of data collection and sampling

Through simple random sampling, households were visited, and household heads were asked for permission to interview the woman. The data was collected using a questionnaire adapted from the Emergency Nutrition Assessment (ENA) household [14] tool with aspects adapted from the WHO signal functions for maternal and child health care tool [15]. The tools were translated into the Runyoro Language, and counter translated back into English to ensure that meaning is not lost. These tools were then pretested in one community in Hoima during the training process of the data collectors to ensure all data collectors can interpret the questions the same way. Any error in the tools were corrected before the actual field data collection.

A team of 10 experienced research assistants recruited from Hoima and who were degree holders, fluent in spoken and written Runyoro and English languages collected the data. They were trained by the investigator for 3 days in ethical data collection, aspects of pregnancy and childbirth, seeking consent from households and the entire methodology for the study.

Following the sampling frame, all mothers with a child presently 0–59 months were included in the study. Those who were currently pregnant, lost their child or only had a child older than 59 months, were excluded in the study. At the household level, a team of two assistants facilitated the data collection process, with one acting as translator. The tool was uploaded on a mobile platform, the Open Data Kit (ODK) [16], and data was collected directly online. A list of the villages in the selected sub-counties was generated and randomized using ENA for SMART software. Households in each cluster (village) were selected using the EPI methodology [17], which is a survey in 30 systematically selected clusters of seven or more children to estimate the immunization coverage.

Five teams with two supervisors collected and checked the quality of the data. To ensure quality of data, the statistician on the team monitored the data on the platform as it was being uploaded. Any errors noticed would quickly be relayed to the field team through the supervisor for correction. Table 1 shows the study variable.

**Table 1:** Study variables

Dependent variables	
**Uptake of ANC services**	
1	ANC in the First Trimester
2	Knowing the 7 Pregnancy Danger Signs
3	Having a birth preparedness and complication readiness plan
4	Taking 90 plus Iron-Folic acid tablets during pregnancy
5	Taking the intermittent Presumptive Treatment (IPT) of malaria
6	Having an HIV Test during ANC
**Essential newborn care**	
1	Knowing the 6 Danger signs of the Newborn
2	Early Initiation of Breastfeeding
3	Practicing Skin to Skin
4	Use of a Clean Birthing Kit
5	Childbirth Assisted by a Skilled Birth Personnel
6	Post-natal care beyond 24 h
**Male involvement in maternal and newborn care**	
1	Assists with home and child care
2	Allocates money to healthcare needs
3	Provides a domestic helper
4	Encourages women to seek healthcare
5	Accompanies women to healthcare
6	HIV counselling and support together
**Independent variables**	
1	Male partner involvement in pregnancy care and immediate newborn care
2	Quality of ANC services
3	Essential newborn care

These variables are defined in the sections below

### Data management and analysis

Data was downloaded from the ODK into Excel. As the data was being collected, each variable was coded independently with a yes or no response. Therefore, where multiple variables contributed to a given indicator, cleaning and alignment were done in Excel. Since the study focused on cross-tabulation of quantitative data through frequencies to deduce percentages, the data was imported into SPSS for analysis. Any missing data meant elimination of that variable in the data going to be analysed since the information followed a continuum of care approach for Maternal Newborn and Child Health [3]*.*

### Measures of social demographic data

Income sources of the household were categorised as permanent or temporary job, or private business with other forms also noted. The availability of the child health and ANC cards served as home-based evidence that particular services had been given to the woman or the baby. Sex and age of the child were also assessed.

### Measures of uptake and quality of ANC services

ANC attendance focussed on the percentage of pregnant women attending four or more visits and having had their initial visit in the first trimester [18]. These parameters were considered sufficient for the uptake of ANC [19].

The quality of Goal-Oriented ANC services [20] variables selected included those that can be acquired from the ANC card or the mother’s passport and those that assesses the mothers’ knowledge. This therefore focussed on the percentages of women who received 5 quality services for the most recent pregnancy. This meant that the women: 1) received iron-folic acid supplements at least once; 2) could recite the 7 danger signs during pregnancy (analysis placed emphasis on seven categories of danger signs [21, 22]: vaginal bleeding, fever, swollen face or extremities, tiredness or breathlessness, headache or blurred vision, convulsions, and reduced or absent foetal movement); 3) was given Fansidar as intermittent presumptive treatment of malaria (IPT) [23]; 4) claimed to have been able to acquire and store the 6 essential commodities of the birth preparedness and complication readiness; and 5) had been tested for HIV [16]. To be able to qualify for this study, a woman had to have a birth preparedness and complication readiness, emergency transport, money saved, sterile blade/scissors for the cord, gloves, cotton wool, and clothes for the baby [24].

### Measures of essential newborn care practices

The essential newborn care practices concerned whether the women received all 7 essential newborn care services as stipulated by the Ministry of Health (MoH) during the birth and immediate newborn period [18] for the most recent birth including if: 1) childbirth was performed at the health facility; 2) childbirth was using a clean birthing kit; 3) childbirth was attended by a doctor, midwife or nurse [25]; 4) placing a baby on skin to skin contact with the women immediately after birth; 5) initiating breasting while still in the labour suite; 6) women and baby stayed at the health facility for 24 h after childbirth; and 7) women claimed to know all six danger signs of the newborn. These newborn danger signs included: difficulty in breathing, not suckling well, high temperature, infectious cord, unclear eyes, and yellow skin colour [26].

### Measures of level of paternal involvement in maternal and newborn care

Analysis of the level of paternal involvement in maternal and newborn care focused on 6 key variables as recommended [27], selected as practices that men could engage in to show participation in maternal and newborn health, based on the woman’s statement that the man: 1) did some household chores, 2) provided money for health care needs, 3) paid a house help because the woman could not engage in heavy household work, 4) reminded and encouraged the woman to attend her hospital visits, 5) accompanied her for at least one visit to the hospital and 6) had an HIV test together.

### A chi square test comparing the 3 key outcome variables and the different income source categories

For the 3 outcome variables, a chi-square test to examine the association between household income source and the key variables was carried out.

### Comparison with national standards

The contribution of the MoH in Uganda to the reduction of maternal and newborn morbidity and mortality is guided by the set targets in the Uganda maternal newborn and child health sharpened plan [3]. At the end of every 5 years of health care implementation, the Ugandan government commissions a nationwide health and demographic status survey [2] to ascertain the level of the services uptake. A comparison of the study results with the national targets set by the sharpened plan and the current national status provided by the Uganda Demographic and Health Survey (UDHS) was also done.

#### Ethical considerations

The conduct of this study was based on clear ethical standards which assured confidentiality, privacy, anonymity and informed consent. The Higher Degrees, Research and Ethics Committee of Makerere University School of Public Health approved the study (protocol number 404). The District Health Officer of Hoima provided the permission to conduct the assessment in their district. The researchers sought informed consent from all the study participants.

## Results

### Respondents’ demographic background

Of the children included in the sample, 50.2% were male and 49.8% were female (see Table 2). The mean age of the children under 5 years included in the study was 28.92 months (SD = 15.60).

**Table 2:** Social demographic information of the participant

Social Demographic Data (***N*** = 691)		
Indicator	N	%
**Sex of the child**		
Female	282	49.8%
Male	284	50.2%
**Age categories of the children**		
0–12 months	108	19.1%
13–24 months	129	22.9%
25–36 months	125	22.2%
37–48 months	120	21.3%
49–59 months	82	14.5%
**Source of household income for the man**		
Permanent job	35	5.1%
Temporary job	167	24.2%
Small business	242	35.0%
Other	247	35.7%
**Presence of essential Health Cards during study**		
Does the child have a Health Card?	525	92.8%
Child card source of information	406	71.5%
Does the woman have an ANC card?	586	84.8%

Regarding the income for the man the households depend on, 35.7% of the household’s source of income was categorized as other, which included depending on seasonal farming and donations from relatives and friends. Even though 29.3% of households depended on a monthly salary as income, only 5.1% were permanent jobs, with the rest being temporary, with uncertainty of continuity. More than a quarter (35%) of the households depended on small businesses.

In total, 92.8% of women had received a Child Health card from the HF, and 71.5% of women presented the card to the study team. Even though 84.8% of the women had an ANC card at the time of study, 5.2% of the women did not have both the ANC card and the Child Health card.

### Uptake and quality of ANC services

Results from Table 3 show that only 24.3% of the women had attended their first ANC in the first trimester, implying that 75.7% of the women in the Hoima District missed out on early uptake of the ANC services offered. Women who had four or more ANC visits during the period of pregnancy were 55.1%. Even though the rest had one, two or three visits, 11.1% of the women had never attended any ANC visit throughout their most recent pregnancy.

**Table 3:** ANC actions during pregnancy (*n* = 691)

Uptake and quality of ANC	N	%
First ANC in the first trimester	168	24.3%
**Number of ANC visits**		
0	77	11.1%
1	10	1.4%
2	34	4.9%
3	189	27.4%
≥ 4	381	55.1%
**Quality of ANC services offered**		
None	9	1.3%
1–2 services	629	91.0%
3–4 services	176	25.5%
All 5 services	3	0.4%

Only 0.4% of women presented evidence of having received all five goal-oriented ANC services. Almost all women (91%) confirmed that they had received at least one to two of the services, with HIV counselling and testing being the most frequently provided service (91%), IPT being the second service (78.9%), and iron-folic acid supplementation contributing only 28.8%.

Goal-oriented ANC scored low because only 13.3% of the women reported having secured all six components of the birth plan. More than half (62.1%) reported having secured at least four to five of the six components mentioned.

Only 3% was able to describe all seven danger signs during pregnancy. It is important to note that 45.3% of women could only mention three to four danger signs, and 23.3% of the women could point out five to six danger signs.

### Essential newborn care practices

Results in Table 4 show that only 0.5% of the women benefited from all 7 ENC practices in their most recent childbirth. A total of 20.1% of the women never practised any of the 7 ENC practices. The majority of the women (79.9%) reported having practised at least one or two ENC, and these were mostly childbirth at the HF and skilled birth attendance.

**Table 4:** Essential newborn care practices (*n* = 209)

Practice of the 7 essential newborn care actions	N	%
None	42	20.1%
1–2	167	79.9%
3–4	158	75.6%
5–6	94	45.0%
7	1	0.5%
**Performance of each of the 7 essential newborn care practices**		
Childbirth at the health facility	167	79.9%
Skilled birth attendant	167	79.9%
Clean birthing kit used	186	89.0%
Skin to skin	199	95.2%
Early initiation of breastfeeding^a^	125	59.8%
Discharge from the HF beyond 24 h	42	20.1%
Recall of 6 Danger signs of the newborn	5	2.4%

^a^to qualify as a woman who did Early initiation of breastfeeding, only those who provided did not provide pre-lacteal feeds were included

The study also examined how each of the seven actions was practised by the women. However, 20% of the women interviewed had not given birth at the HF, and the same percentage was not assisted by a skilled birth attendant. For those who delivered at the HF, 95.2% practised skin to skin contact, 89% used a clean birthing kit, but only 59.8% of the women reported having initiated breastfeeding within the first hour of life. For length of stay at the HF after delivery, 24.9% of the women were discharged from the HF within 6 h after birth, and another 34.9% of the women were discharged within 24 h. Less than a quarter (20.1%) of women who delivered at the HF were discharged beyond 24 h after delivery.

Only 2.4% of women understood and recalled all the six danger signs of the newborn. Individual examination of the danger signs was not very successful, with identification of an infectious cord scoring 14.8%, followed by baby not suckling well with 13.4%, and identification of yellow skin scoring the lowest with 7.7%.

### Male involvement in maternal and newborn care

Results from the study as shown in Table 5 indicate that only 11.1% of the women declared that the man supported them with all 6 aspects. At least 46.7% reported that the man was involved in one to three aspects during pregnancy and the immediate newborn period. It is important to note that 21.1% of the women reported that their male partners did not provide any support at all. Of the 6 components, allocation of money to the woman for healthcare needs stood out as the most widely practised behaviour (63.7%). The study further revealed that 52.5% of women had their male partners take time off from their schedules to assist with household work and child care as the pregnant woman rested, and 49.2% of the woman confirmed that they had a household helper during the newborn period, paid for by the male counterpart.

**Table 5:** Male involvement in maternal and newborn care

Male involvement in the six aspects of maternal and newborn care (691)	N	%
None	146	21.1%
1–3	323	46.7%
4–5	145	21.0%
6	77	11.1%
**Practices of male partner in pregnancy care and immediate newborn care**		
Assists with home and child care	363	52.5%
Allocates money to healthcare needs	440	63.7%
Provides a domestic helper	340	49.2%
Encourages women to seek healthcare	276	39.9%
Accompanies women to healthcare	255	36.9%
HIV counselling and support together	181	26.2%

Of the 6 components, 3 focused on practices that male counterparts should do to contribute to goal-oriented ANC care. Women reported that only 39.9% of their male counterparts encouraged them to actually seek care focusing on the ANC visits. This was further confirmed by the low levels of male counterparts (36.9%) accompanying their pregnant women to ANC. Even though the MoH guidelines encourage couple counselling and testing for HIV, to accelerate the reduction of woman to child transition of HIV, only 26.2% of male counterparts took an HIV test with their pregnant women during ANC.

### Chi square test comparing the 3 key variables and the different income source categories

Table 6 shows the percentages of participants that meet all indicators for the three key variables, according to household income source. The chi-square tests show that household income source significantly predicts male involvement, with males being more often involved in the pregnancy and newborn care if the income comes from a small business or temporary job, compared to households where the income comes from a permanent job or other sources.

**Table 6:** Key variables according to source of household income

Chi-Square Tests against House hold source of livelihood		Numbers and Percentages		Significance (***p***-value)^**a**^
		No	Yes	
**Quality of ANC care**	Permanent job	34(4.9%)	1 (0.1%)	0.115
	Temporary Job	166 (24.0%)	1 (0.1%)	
	Small Business	241 (34.9%)	1 (0.1%)	
	Others	247 (35.7%)	0 (0.0%)	
**Essential Newborn care**	Permanent job	12 (5.7%)	0 (0.0%)	0.350
	Temporary Job	48 (23.0%)	1 (0.5%)	
	Small Business	69 (33.0%)	0 (0.0%)	
	Others	79 (37.8%)	0 (0.0%)	
**Male involvement in maternal and Newborn Care**	Permanent job	30 (4.3%)	5 (0.7%)	< 0.001
	Temporary Job	136 (19.5%)	31 (4.5%)	
	Small Business	202 (29.2%)	40 (5.8%)	
	Others	246 (35.6%)	1 (0.1%)	

^a^ Results of chi-square test

### Performance of Hoima District compared with national requirements

**Fig. 1:**
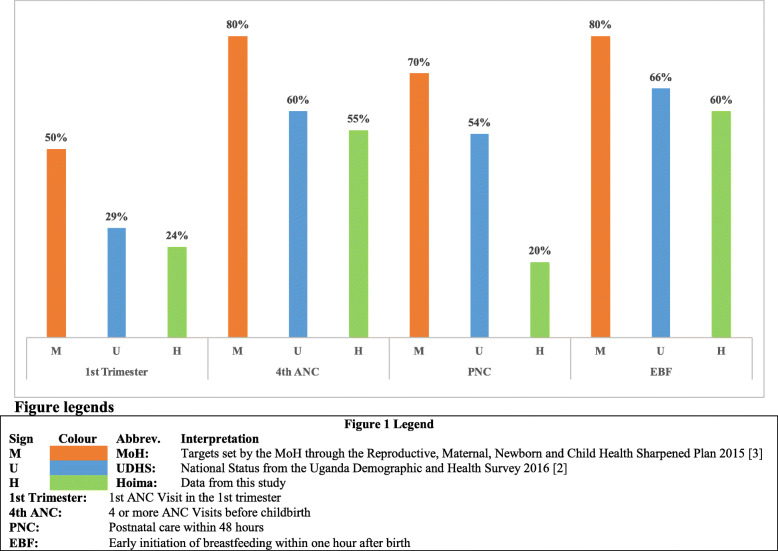
Hoima District key indicators against national performance and targets. M. MoH: Targets set by the MoH through the Reproductive, Maternal, Newborn and Child Health Sharpened Plan 2015 [3]. U. UDHS: National Status from the Uganda Demographic and Health Survey 2016 [2]. H. Hoima: Data from this study. 1st Trimester: 1st ANC Visit in the 1st trimester. 4th ANC: 4 or more ANC Visits before childbirth. PNC: Postnatal care within 48 h. EBF: Early initiation of breastfeeding within 1 h after birth

As shown in Fig. 1, in the Hoima District, earlier first attendance at ANC is 26% below the national target, but only 5% below the national achievement (UDHS), meaning that most woman attend their first ANC visit after 12 weeks of gestation. The Hoima District has a low 4th ANC attendance percentage, which is 5% below the national average and 25% below the Ugandan national target. The Hoima District PNC practices within the first 48 h after birth are 50% below the national target and 34% below the national practice. The Hoima District early initiation of breastfeeding is 20% below the national target but only 6% below the national average.

## Discussion

This study aimed to assess the maternal and newborn healthcare status at the household level, focusing on lifesaving services uptake through the continuum of care approach from pregnancy to the completion of the newborn timeframe. Uptake of ANC services focused on identifying early ANC attendance, which was low and 4 or more ANC visits, which stood at little more than 50% in the Hoima District. Results further showed that almost none of the women benefited from all 7 ENC practices in their most recent childbirth, with 20.1% of the women never practising any of them. Paternal involvement was very low, mostly women declaring that the male partner supported them with all six aspects of the study focus, on women reporting that their male partners did not provide any support. These results are worrying, and the Hoima District is at great risk of not achieving the MNH-related SDGs [28].

The study revealed that even though the Ugandan government has set targets and promoted goal-oriented ANC, women in the Hoima District generally still do not start their ANC in the first trimester [21, 29]. Results from the study show that less than a quarter of the women had their first ANC in the first trimester, implying that 65.7% of the women missed out on an early uptake of the ANC services offered. This could perhaps be attributed to the high levels of teenage pregnancy in the Hoima District [30], which leads to unwanted pregnancies that attract limited care-seeking. Teenage pregnancy is considered negative by the community, and therefore the teenage women’ ability to be supported to seek care early is limited by practices like hiding the pregnancy, absent male partners and unwilling parents [31]. This means goal-oriented care starts late, presenting a missed opportunity for both the woman and the unborn child. If a woman starts the first ANC late, she will not be able to attend four or more ANC visits [32].

The quality of ANC services offered in Hoima was alarmingly poor, based on the performance of 5 components involved. This is attributed to its geographical location; it is a border district to the Democratic Republic of the Congo and constantly has an influx of refugees from there. Due to such circumstances, it is challenging to keep good-quality service providers at the health facilities to offer goal-oriented ANC care. The quality of ANC a woman receives during pregnancy is crucial to both the child and the woman’s life. The focus on services offered is to ensure that the growth of the child is facilitated and to promote the recommended birth preparedness and childbirth care, which in turn fosters a positive impact on ENC [33].

Results showed that almost none of the women benefited from all seven ENC practices during their most recent childbirth. This is quite low considering that Berhe et al. (2017) revealed that 26.7% of their participants fulfilled essential newborn care practices in Ethiopia [34]. Tesfaye Yitna Chichiabellu et al. found a roughly similar trend in the southern part of Ethiopia, with the prevalence of essential newborn care practice being 24% [35]. The coverage of ENC practices in the current study was low. ANC visits, counselling about birth preparedness, PNC, and knowledge about newborn danger signs are important predictors of ENC practice in this study. Hoima District needs to focus on community-based interventions that promote childbirth at the health facility, using a Maama Kit at childbirth for clean birthing, skilled birth attendance, performance of skin to skin contact, early initiation of breastfeeding, length of stay at the HF after childbirth, and the woman’s knowledge of danger signs for the newborn since these are the practices that were included in the study.

Almost none of the participating women understood and recalled all six danger signs of the newborn. Individual examination of the danger signs was not very successful. This is not surprising for the Hoima District, because for women to understand the danger signs, there has to be vigorous counselling support both at the health facility and in the community through the community health workers. However, since most of Hoima District’s population consists of refugees, the visibility of the community health workers who normally facilitate household counselling is minimal and ever changing. Early detection of newborn illness is a step towards improving newborn survival. If the women are taught to identify danger signs of the newborn early enough, there would probably be a significant reduction in the babies who die in the first 28 days of life since most of them die of preventable causes [36].

Results from the study showed that a limited number of the women declared that the male partners supported them with all six aspects, indicating that paternal involvement is a big challenge in maternal and child health care. Men play the role of gatekeepers to healthcare, they are the primary decision-makers in Ugandan households and thus directly affect their partner’s and children’s health. Their decisions impact the utilization of resources and access to health care services, the availability of nutritious food and the women’s workload. Therefore, leveraging the roles of men to positively influence decision-making around MNH is important for improving the health and wellbeing of women and newborns [37].

This study had some limitations. An important one was recall bias for variables included in the study, as the women needed to recall what happened up to 5 years before the study. A number of practices and services included in the study were ascertained by the availability of the child health card or an ANC card, but for women (5.2%) who did not have both of them, the information provided based on recall further contributed to the recall bias. In addition, maternal age was not included in the data collection. Results from the study provide an opportunity to strengthen guided maternal newborn and child health-focused implementation and present suggestions for further studies.

This study suggested recommendations as follow: With this low uptake and quality of ANC, there is a need to invest in community-based interventions that facilitate the implementation of interventions to promote birth preparedness and complication readiness and understanding the danger signs during pregnancy. The actions for essential newborn care happen in the immediate newborn period and are supposed to be facilitated at the health facility. Interventions geared towards ensuring essential newborn care need to be accelerated at both the household and health facility levels, to ensure that women benefit from these practices. With the current low level of male support, community-based initiatives geared towards dialoguing with men would go a long way to improving male participation in pregnancy and childbirth care.

## Conclusion

The study reveals poor maternal and newborn practices through the continuum of care, starting from ANC, skilled birth attendance to newborn care during the postnatal period. With such poor results, it is not surprising that Hoima is sixth of 10 districts that have the highest numbers of deaths due to maternal mortality in Uganda. If this situation does not change, Hoima may not contribute substantially to the targets of the sustainable development goals for Uganda. Further still household income is a significant predictor of male involvement.

## Data Availability

The raw data will be made available upon reasonable request from the corresponding author.

## Abbreviations

ANC: Antenatal care
ENC: Essential newborn care
HF: Health facility
IPT: Intermittent presumptive treatment of malaria
MNH: Maternal and newborn health
MoH: Ministry of Health
ODK: Open data kit
PNC: Postnatal care
SDGs: Sustainable development goals
UDHS: Uganda Demographic and Health Survey

## References

[1] World Health Organisation (2018). Maternal mortality updates.

[2] Uganda Bureau of Statistics. The Uganda Demographic and Health Survey (UDHS). Kampala: UBOS; 2016.

[3] Ministry of Health Uganda. The reproductive Health, maternal, Newborn and Child Health Sharpened plan. Kampala: MoH; 2013.

[4] World Health Organisation (2016). New guidelines on antenatal care for a positive pregnancy experience.

[5] Geoffrey B, Gilbert K, Anita K (2017). Bridging the gap for maternal newborn and child health human resources in rural Uganda: experiences and lessons learnt from world vision east African maternal newborn and child health project implementation, Kitgum District. Nur Primary Care.

[6] World Health Organisation (2018). Infant and young child feeding.

[7] World Health Organisation (1917). Thermal Protection of the Newborn.

[8] Kabwijamu L (2016). Newborn care practices among adolescent mothers in Hoima District, Western Uganda. Plos One.

[9] Tokhi M, Comrie-Thomson L, Davis J, Portela A, Chersich M, Luchters S (2018). Involving men to improve maternal and newborn health: a systematic review of the effectiveness of interventions. Plos One.

[10] Sharma S, Kc B, Khatri A (2018). Factors influencing male participation in reproductive health: a qualitative study. J Multidiscip Healthc.

[11] Rahman AE, Perkins J, Islam S (2018). Knowledge and involvement of husbands in maternal and newborn health in rural Bangladesh. BMC Pregnancy Childbirth.

[12] Hioma district. From Wikipedia, the free encyclopedia. Uganda: MoLG; 2018.

[13] Uganda Bureau of Statistics. The Population of The Regions of the Republic of Uganda And All Cities And Towns of More Than 15,000 Inhabitants. Kampala: UBOS; 2014.

[14] Software for Emergency Nutrition Assessment. ENA,2016.

[15] Gabrysch S, Civitelli G, Edmond KM, Mathai M, Ali M (2012). New signal functions to measure the ability of health facilities to provide routine and emergency newborn care. Plos Med.

[16] Open Data kit. ODK, 2018.

[17] Kristof Bostoen and Zaid Chalabi , Optimization of household survey sampling without sample frames. Int J Epidemiol. 2006;35(3):751–5. 10.1093/ije/dyl019 16481364

[18] The Republic of Uganda. Roadmap for Accelerating the Reduction of Maternal and Neonatal Mortality and Morbidity in Uganda. Kampala: MoH; 2015.

[19] Ministry of Health Uganda. Health sector strategic and investment plan 2015/16–2019/20. Kampala: MoH; 2015.

[20] Carroli G, et al,2001, WHO systematic review of randomized controlled trials of routine antenatal care. Lancet. 2001;357(9268):1565–70. 10.1016/S0140-6736(00)04723-1 11377643

[21] Ministry of Health Uganda. Uganda Clinical Guidelines. National Guidelines for Management of Common Conditions. Kampala: MoH; 2016.

[22] Ministry of Health Uganda. Village Health Team. A handbook to improve health in communities. Kampala: MoH; 2010.

[23] Ministry of Health Uganda. Uganda Malaria Reduction Strategic Plan. Kampala: MoH; 2014.

[24] Ministry of Health Uganda. Maama Kit: Making childbirth clean and safer. Kampala: MoH; 1997.

[25] Healthy Newborn Network. Essential newborn care. Massachusetts: HNN; 2019.

[26] Alex-Hart B (2014). 2014, mothers’ recognition of newborn danger signs and health seeking behaviour. Niger J Paed.

[27] Suzanne Kiwanuka, Male Involvement in Maternal Health: Helpful or Harmful? Kampala: MHTF; 2015.

[28] Government of Uganda. REVIEW REPORT ON UGANDA’S READINESS FOR IMPLEMENTATION OF THE 2030 AGENDA. Kampala: GoU; 2016.

[29] Šimundić A-M (2013). 2014, Bias in research. Biochem Med.

[30] Kirungi Kasozi G, Kasozi J, Pio Kiyingi F, Musoke M (2019). School-based sexual and reproductive health Services for Prevention of adolescent pregnancy in the Hoima District, Uganda: Cluster Randomized Controlled Trial. Methods Protoc.

[31] Bwalya BC, Sitali D, Baboo KS, Zulu JM (2018). Experiences of antenatal care among pregnant adolescents at Kanyama and Matero clinics in Lusaka district, Zambia. Reprod Health.

[32] Ewunetie AA, Munea AM, Meselu BT (2018). DELAY on first antenatal care visit and its associated factors among pregnant women in public health facilities of Debre Markos town, North West Ethiopia. BMC Pregnancy Childbirth.

[33] Larsen A, Cheyip M, Aynalem G (2017). Uptake and predictors of early postnatal follow-up care amongst women-baby pairs in South Africa: results from three population-based surveys, 2010-2013. J Glob Health.

[34] Berhe M, Medhaniye AA, Kahsay G, Birhane E, Abay M (2017). Essential neonatal care utilization and associated factors among women in public health facilities of Aksum Town, North Ethiopia, 2016. Plos One.

[35] Chichiabellu TY, Mekonnen B, Astawesegn FH (2018). Essential newborn care practices and associated factors among home delivered mothers in Damot pulasa Woreda, southern Ethiopia. Reprod Health.

[36] Ministry of Health (2007). Situation analysis of newborn health in Uganda: current status and opportunities to improve care and survival.

[37] Maluka SO, Peneza AK (2018). Perceptions on male involvement in pregnancy and childbirth in Masasi District, Tanzania: a qualitative study. Reprod Health.

